# Paroxysmal supraventricular tachycardia as first manifestation of right atrial hemangioma during endovascular treatment of intracranial arteriovenous fistulas

**DOI:** 10.18632/oncotarget.3781

**Published:** 2015-04-27

**Authors:** Francesca Spanò, Alberto Cereda, Antonella Moreo, Edgardo Bonacina, Angelica Peritore, Alberto Roghi, Cristina Giannattasio, Patrizia Pedrotti

**Affiliations:** ^1^ Department of Cardiology A De Gasperis, Cardiology 4, Echocardiography Laboratory, Niguarda Cà Granda Hospital, Milano, Italy; ^2^ Bicocca University, Science of Health Department, Milano, Italy; ^3^ Pathology Laboratories, Niguarda Cà Granda Hospital, Milano, Italy; ^4^ Department of Cardiology A De Gasperis, Cardiology 4, Cardiovascular Magnetic Resonance Unit, Niguarda Cà Granda Hospital, Milano, Italy; ^5^ Department of Cardiology A De Gasperis, Cardiology 4, Niguarda Cà Granda Hospital, Milano, Italy

**Keywords:** paroxysmal supraventricular tachycardia, atrial mass, mixed type cavernous-capillary hemangioma, cardiac imaging, cerebral fistulas

## Abstract

We report the description of a cardiac mass occupying almost the entire right atrium in a young man who developed paroxysmal supraventricular tachycardia during endovascular treatment of intracranial arteriovenous fistulas. The mass was detected at echocardiographic examination, its tissue characteristics were defined with cardiac magnetic resonance and it was successfully surgically removed. The histopathological findings were consistent with a mixed type cavernous-capillary hemangioma of the heart. The intriguing co-existence of cardiac hemangioma and cerebral arteriovenous fistulas, to the best of our knowledge, has not been previously reported in English Literature.

## INTRODUCTION

Hemangiomas are mostly benign tumors consisting of blood vessels, frequently localized in the skin and subcutaneous tissues. Their localization in the heart is very rare, with an incidence of 5% among benign cardiac tumors in surgical series. Fewer than 30 cases of right atrial cavernous hemangiomas have been reported in English literature.

Although most hemangiomas are asymptomatic, symptoms may develop depending on the location, size and mobility of the tumor; arrhythmias, pericardial effusion, coronary insufficiency, heart failure and sudden cardiac death have been reported [1–3].

## CASE REPORT

**Figure 1:**
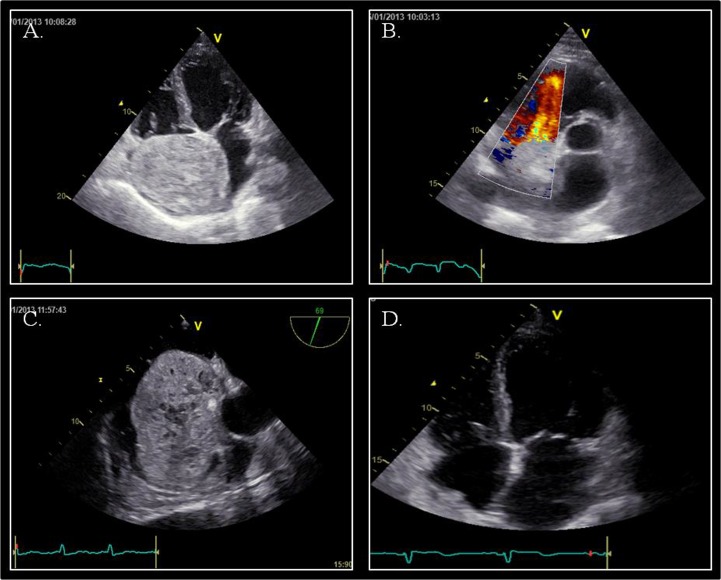
Echocardiography Panel **A.** Apical four-chamber view showing a voluminous mass adhering to inter-atrial septum. Panel **B.** Parasternal short-axis view: turbulence in the right ventricular inflow tract; no signs of right outflow obstruction. Panel **C.** Mid-oesophageal aortic valve long axis view (70°): atrial mass with evidence of parenchymatous density and multiple echolucent structures occupying almost the whole right atrium with no evidence of caval veins obstruction. Panel **D.** Apical four-chamber view after surgical removal of the tumor mass.

A 47-year old male patient, with past unremarkable medical history, developed 7th right cranial nerve palsy, which was investigated with computed tomography and magnetic resonance of the head, followed by cerebral angiography showing dural arteriovenous fistulas (dAVFs) of the right transverse-sigmoid sinus. The patient was hospitalized to perform endovascular embolization of dAVFs. During the procedure he developed rapid paroxysmal supraventricular tachycardia (PSVT); the patient was sedated and intubated for the procedure in course and there were no signs of hemodynamic compromise. Vagal maneuvers and adenosine were ineffective and sinus rhythm was restored with intravenous amiodarone. The patient was subsequently questioned in search for symptoms suggestive of underlying cardiac disease, but he denied any history of palpitations, syncope, and dyspnea or chest pain. Transthoracic echocardiography (TTE) revealed a huge round shaped mass with parenchymatous density and multiple echolucencies occupying almost the whole right atrium (Figure 1), with no significant right inflow obstruction, although turbolent flow was seen. For a better evaluation, TTE with ultrasound contrast SonoVue^®^ (Video 1) and transesophageal echocardiography (TOE) were performed. The mass (9 × 7.5 cm) adhered to the inter-atrial septum and showed signs of vascularity after injection of echo contrast. There was no evidence of local invasion or compressing phenomena to adjacent structures and there were no signs of right inflow tract and caval veins obstruction.

**Video 1:**
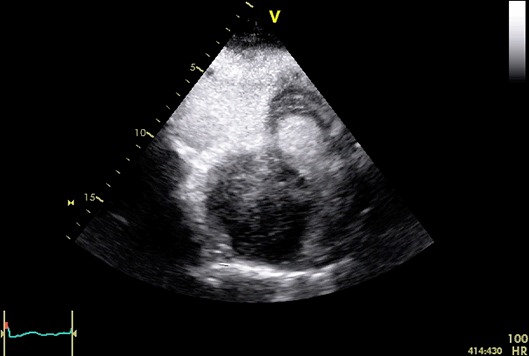
Transthoracic echocardiography, off-axis view of the right atrium, acquired at the end of contrast infusion, showing perfusion of the mass

The right ventricle was slightly dilated with preserved systolic function and there was no tricuspid regurgitation. Left cardiac chambers were normal.

**Figure 2:**
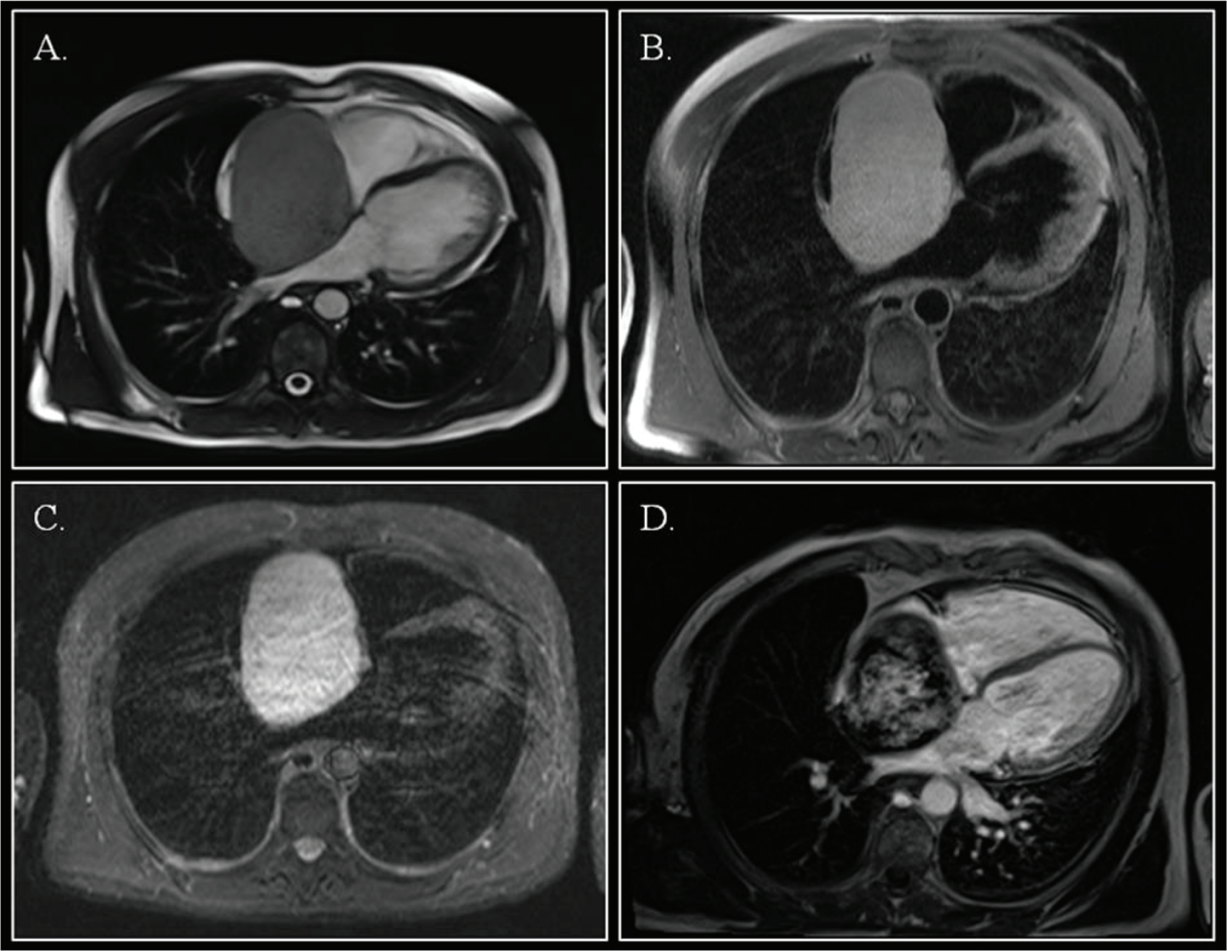
Cardiac magnetic resonance Steady-state free precession cine imaging demonstrated a right atrial mass with a large base of implant on the inter-atrial septum. (Panel **A.**; still frame, off-axis four-chamber view). The mass was hyperintense on T1 images with fat saturation (Panel **B.**), and on T2-weighted STIR images (Panel **C.**). After gadolinium injection, inhomogeneous late gadolinium enhancement was evident (Panel **D.**).

Cardiac magnetic resonance imaging (CMR) showed a capsulated cardiac mass with a large base of implant on the interatrial septum with clearly visible cleavage planes (Figure 2; Video 2). The mass was hyperintense on T1 and T2-weighted images, showed intense enhancement on first-pass perfusion (Video 3) after administration of gadolinium and inhomogeneous delayed enhancement. Blood investigations, thrombophilic screening, neoplastic markers and autoimmune assays were negative. Coronary angiography showed unobstructed coronary arteries, without signs of compression in the atrio-ventricular groove; vessels originating from the right coronary artery fed the tumor.

**Video 2:**
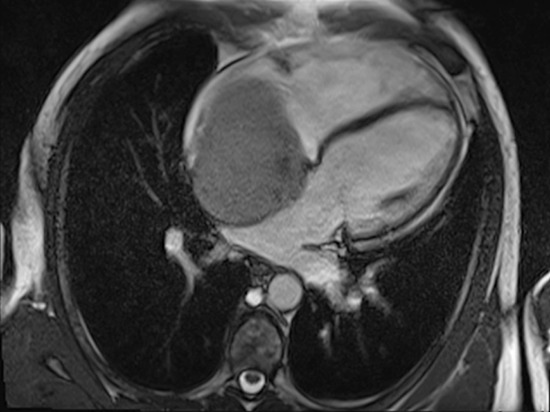
CMR, four chamber view SSFP cine image, showing a huge mass occupying the right atrium, without right ventricular in-flow obstruction

**Video 3:**
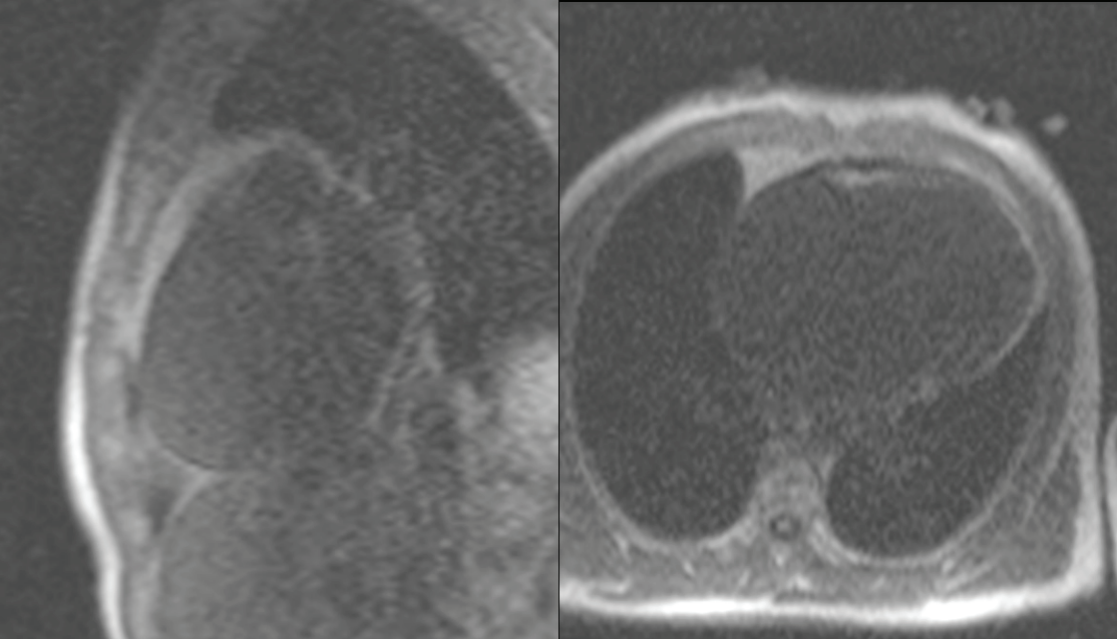
CMR, short axis (left panel) and four chamber views (right panel), showing mass vascularization at first pass perfusion imaging

**Figure 3:**
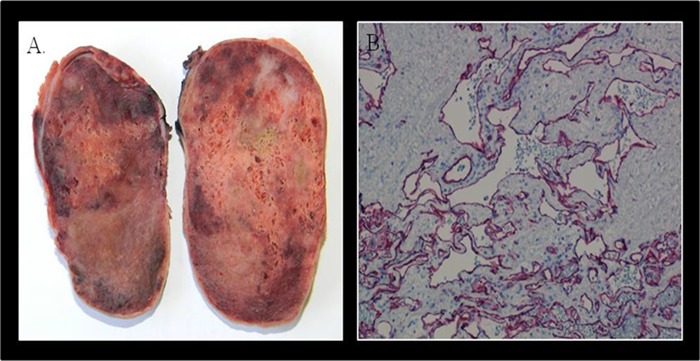
Pathology The excised specimen revealed a well defined encapsulated nodular fleshy mass, weighting 120 gram and measuring 8.5 × 5.5 × 5 cm, with a spongy consistency and reddish brown color (Panel **A.**). Microscopic examination showed features of a mixed type cavernous-capillary hemangioma. The immunohistochemical profile was CD34 positive, CD31 positive (Panel **B.**), factor VIII positive, alpha actin positive, cytokeratin negative

The right atrial mass was surgically removed “en bloc” and the hystopathological diagnosis was consistent with “mixed type cavernous-capillary hemangioma” with no evidence of malignant cells; resection margins were free of neoplastic tissue. The immunohystochemical profile (CD34 +, CD31 +, factor VIII +, alpha actin +, cytokeratin −) was consistent with cardiac hemangioma (Figure 3).

Postoperative course was uneventful and follow-up TTE at one month was normal with no signs of recurrence. Despite the successful removal of the cardiac mass, five months later the patient died for hemorrhagic stroke caused by cerebral arteriovenous fistula rupture.

## DISCUSSION

Primary cardiac tumors are a rare entity and account for 5-10% of all cardiac tumors [4,5]; 25% of them are malignant and 75% of these are sarcomas. The relative incidence of hemangiomas is 2.8% [6].

Most common symptoms of hemangioma are dyspnea, palpitations, chest pain and arrhythmias. There are only a few reports of paroxysmal supraventricular tachycardia and neurological manifestations due to embolization and there are a few reports of cardiac hemangiomas presenting as bloody pericardial effusion with fatal tamponade [7].

Multimodality imaging plays a key role in cardiac tumor differential diagnosis and in surgical resection planning. The differentiation of a benign tumor from a malignant one is essential in planning the subsequent management [8]. Clinical echocardiography is often the initial evaluation: it localizes the mass, gives information about size, mobility, compression of adjacent structures and it allows differentiation between solid and liquid mass; contrast injection can identify mass vascularization [8]. CMR can further depict tumor characteristics: angiosarcomas can present as single or multiple masses, without a clear cleavage plane; tissue signal intensity is very heterogeneous, for the presence of necrotic and hemorrhagic areas; first pass perfusion shows vascularization and post-contrast enhancement is inhomogeneous. Myocardial and pericardial infiltration with pericardial effusion occurs [9,10]. Metastatic involvement of the lungs is a frequent finding and can be better defined with computed tomography [8]. At CMR, differentiation of hemangioma from myxoma is more challenging, as both tumors show high signal intensity on STIR-T2 images and inhomogeneous post-contrast enhancement, although hemangioma shows higher vascularization on first pass images and the most frequent location of myxoma is the left atrium [9]. From a pathological point of view, hemangiomas are classified into cavernous, capillary and arteriovenous. Myxoma and angiosarcoma should be considered in the differential diagnosis; some low-grade angiosarcomas may be difficult to differentiate from hemangiomas.

In a review of 56 cases of cardiac hemangiomas, 36% were found in the right ventricle, 34% in the left ventricle, and 23% in the right atrium, and the rest on the interatrial septum and in the left atrium [9]. Heart valve hemangioma is a rare finding, although the case report of a hemangioma completely replacing the septal leaflet of the tricuspid valve has been reported by our group [12].

An intriguing co-existence of atrial myxoma and cerebral cavernous malformations has been described and the association of cerebral vascular malformations with extracranial/mesenchimal anomalies could be coincidental or caused by a common unknown genetic disorder [13,14].

To the best of our knowledge, there are no previous descriptions reporting cardiac hemangioma in a patient with dAVF.

## CONCLUSION

Cardiac imaging played an essential role in the detection and evaluation of cardiac hemangioma, providing valuable information regarding size, location, morphology, and the relationship to adjacent structures, thus allowing surgical planning of mass resection. To the best of our knowledge, this is the first reported case of the association of cardiac hemangioma and cerebrovascular malformation
